# A labelled dataset of healthy and diseased common bean (*Phaseolus vulgaris*) from Tanzania

**DOI:** 10.1016/j.dib.2026.112909

**Published:** 2026-05-31

**Authors:** Neema Mduma, Hudson Laizer

**Affiliations:** a School of Computational and Communication Sciences and Engineering, The Nelson Mandela African Institution of Science and Technology, P.O Box 447, Tengeru, Arusha, Tanzania; b School of Life Sciences and Bioengineering, The Nelson Mandela African Institution of Science and Technology, P.O. Box 447, Tengeru, Arusha, Tanzania

**Keywords:** Phaseolus vulgaris, Image dataset, Machine learning, Deep learning, Crop disease detection, Bean anthracnose, Bean rust

## Abstract

Common bean (*Phaseolus vulgaris*) is an important food and cash crop in Tanzania, where it contributes to household nutrition and income among smallholder farmers. Its production is, however, constrained by diseases such as bean rust and bean anthracnose, which can cause substantial yield losses and reduce crop quality. This article presents a labelled image dataset of common bean leaves collected under field conditions in Northern Tanzania to support research and application development in computer vision, machine learning and digital crop health. The dataset comprises 155,842 labelled multi-season images belonging to three classes: healthy leaves, bean rust and bean anthracnose. Images were acquired from farms in Arusha, Kilimanjaro and Manyara regions using smartphone cameras and were subsequently reviewed and validated with support from agricultural extension officers and plant pathologists to improve annotation reliability. The dataset is organized into class-specific compressed files and is publicly available through the Zenodo repository. By providing a large field-based image resource captured under variable real-world conditions, this dataset can support the development, training and evaluation of image-based models for common bean disease identification.

Specifications Table

**Table:** 

Subject	Computer Sciences
Specific subject area	Computer Vision for Detection of Common Bean Diseases (Bean Rust and Bean Anthracnose)
Type of data	Image Raw
Data collection	Data were collected using Samsung Galaxy M13 smartphones equipped with 13-megapixel cameras. The Open Data Kit (ODK) application was installed on the devices to capture common bean leaf images directly from the field. The raw images were categorized into three classes: healthy, bean rust and bean anthracnose. The data collection process involved researchers and farmers, while agricultural extension officers and plant pathologists conducted quality assurance and validation.
Data source location	• Institution: The Nelson Mandela African Institution of Science and Technology, Tanzania Agricultural Research Institute • City/Town/Region: Arusha • Country: Tanzania
Data accessibility	Repository name: Zenodo Data identification number: doi:10.5281/zenodo.15685315 Direct URL to data: https://zenodo.org/records/15685315

## 1 Value of the Data

• This dataset provides a large, field-based collection of labelled common bean leaf images representing healthy leaves, bean rust and bean anthracnose from major bean-growing areas of Northern Tanzania. Because the images were captured under real farm conditions rather than in controlled laboratory settings, the dataset reflects practical variation in background, lighting, leaf orientation, symptom expression and image quality. This makes it practically useful for developing models intended for real-world agricultural applications.
• The dataset is valuable to researchers working in computer vision, machine learning, digital agriculture and plant pathology. It offers a locally relevant resource for training, validating and benchmarking image-based disease recognition models for common bean. Since many publicly available plant disease datasets are developed under controlled conditions or from regions outside East Africa, this dataset helps address the need for context-specific data that better represent the production environments in which diagnostic tools are expected to operate.
• The dataset can support a wide range of analytical and applied tasks such as image classification, comparative testing of deep learning architectures, transfer learning, model generalization studies and the development of mobile or web-based decision-support tools for disease diagnosis. It may also be useful for studies examining the effect of field variability on model performance, especially where robust disease detection under non-standardized conditions is required.
• This dataset can serve as a baseline resource for integrating additional disease classes, broader geographical coverage, symptom severity levels or complementary metadata in future studies. As an openly accessible dataset with a persistent repository link, it supports transparency, reproducibility and wider reuse in both methodological research and practical agricultural innovation.

## 2 Background

Common bean (*Phaseolus vulgaris*) is among the most widely cultivated grain legumes in Tanzania and across Sub-Saharan Africa, playing an important role in the livelihoods of smallholder farmers [1,2]. It is widely consumed in both rural and urban households and serves as an accessible source of dietary protein, minerals and income [3]. In many farming systems, common bean is cultivated either as a sole crop or in association with other staple crops, making it an important component of household nutrition and local market systems [4]. Its contribution to food security is especially important in regions where animal protein is less accessible or less affordable [5]. Despite these, common bean productivity is frequently constrained by pests and diseases, which continue to reduce yields and affect crop quality [6,7]. Among the major foliar diseases, bean rust and bean anthracnose are of particular concern because of their widespread occurrence and their potential to cause substantial production losses under favourable conditions [8]. These diseases affect leaf health and plant vigor and, when not managed in time, may reduce both yield and market value [9]. Their effects are often more serious in smallholder production systems where routine disease monitoring and timely technical support may be limited [10,11].

Accurate recognition of plant diseases at an early stage is essential for effective crop management. In practice, diagnosis in many farming areas relies mainly on visual inspection by farmers, extension officers or local experts [12,13]. This approach can be constrained by limited access to specialist knowledge, variation in field conditions and overlap in visible symptoms across diseases or stress factors [14]. These limitations have increased interest in digital approaches that can assist with crop health diagnosis in a faster and more standardized way [15]. Image-based diagnosis supported by artificial intelligence has emerged as a promising area in agricultural research, with computer vision methods showing strong potential for identifying visible disease symptoms from leaf images [16,17]. The usefulness of these methods, however, depends greatly on the quality, quantity and representativeness of the training data used to develop them [18]. Datasets collected in controlled settings may not adequately reflect the variability encountered in real farm environments, where differences in lighting, background, camera angle, symptom severity and field conditions can affect model performance [19]. For this reason, there is a growing need for datasets collected under natural production conditions and from locations where these tools are intended to be applied.

This work presents a labelled dataset of common bean leaf images collected from farms in Arusha, Kilimanjaro and Manyara regions of Tanzania. The dataset includes healthy leaves together with leaves showing symptoms of bean rust and bean anthracnose. It was developed to provide an openly accessible image resource that reflects field-based conditions and that can support future work in machine learning, computer vision and digital crop health applications. By describing the dataset and its organization, this article contributes to the availability of reusable data for common bean disease research and related technological development.

## 3 Data Description

This dataset consists of common bean leaf images collected from farms in Tanzania, including both healthy samples and those affected by bean rust and bean anthracnose. It was developed to support research on disease diagnosis and to enhance crop productivity through data-driven approaches. The dataset is suitable for a range of computer vision tasks, including image classification and object detection. All images are organized in compressed (.zip) files based on class categories. The bean anthracnose class is provided in “anthra.zip” containing 13,542 images. Bean rust images are distributed across two files, “rust1.zip” with 10,300 images and “rust2.zip” with 10,307 images. Healthy common bean leaf images are divided into eleven files, where “healthy1.zip” to “healthy10.zip” each contain 11,000 images, and “healthy11.zip” includes 11,693 images. In total, the dataset comprises 155,842 labeled leaf images.

As shown in Table 1, the dataset exhibits class imbalance, with a substantially larger number of healthy images compared to diseased classes, particularly bean anthracnose. While this reflects real-world field conditions, it may influence model training and evaluation. Users of the dataset may therefore need to consider appropriate strategies such as resampling, class weighting or the use of balanced evaluation metrics when developing machine learning models.

**Table 1:** Number of common bean leaf images per categories.

Class name	Number of images
Healthy	121,693
Bean rust	20,607
Bean anthracnose	13,542

Each ZIP archive contains images in JPEG (.jpg) format. In addition, image-level metadata is embedded within the files in EXIF format. This includes information such as image dimensions, device make and model, capture date and time and camera acquisition parameters (e.g., exposure time and focal length). Where available, location-related metadata may also be present. This embedded metadata enables users to access both visual and contextual information directly from the images, supporting reproducibility and extended analyses.

This dataset is distinct from existing image datasets in both domain and application. Unlike general-purpose or non-agricultural datasets (e.g., those used for tasks such as floor count estimation), it focuses specifically on common bean (*Phaseolus vulgaris*) leaves under real field conditions. The images capture natural variability in lighting, background and disease presentation, which enhances their relevance for practical agricultural applications. Furthermore, the dataset provides labeled examples of key diseases affecting common bean production, making it suitable for developing and evaluating machine learning models for plant disease detection and decision support in digital agriculture.

Although this article does not include model development or performance evaluation, the dataset has been structured to support machine learning applications such as crop disease detection. The availability of labeled images across multiple classes, along with real field variability, makes it suitable for training and benchmarking computer vision models. Users may apply standard machine learning and deep learning approaches to evaluate classification performance using this dataset.

## 4 Experimental Design, Materials and Methods

### 4.1 Field data collection

**Fig. 1:**
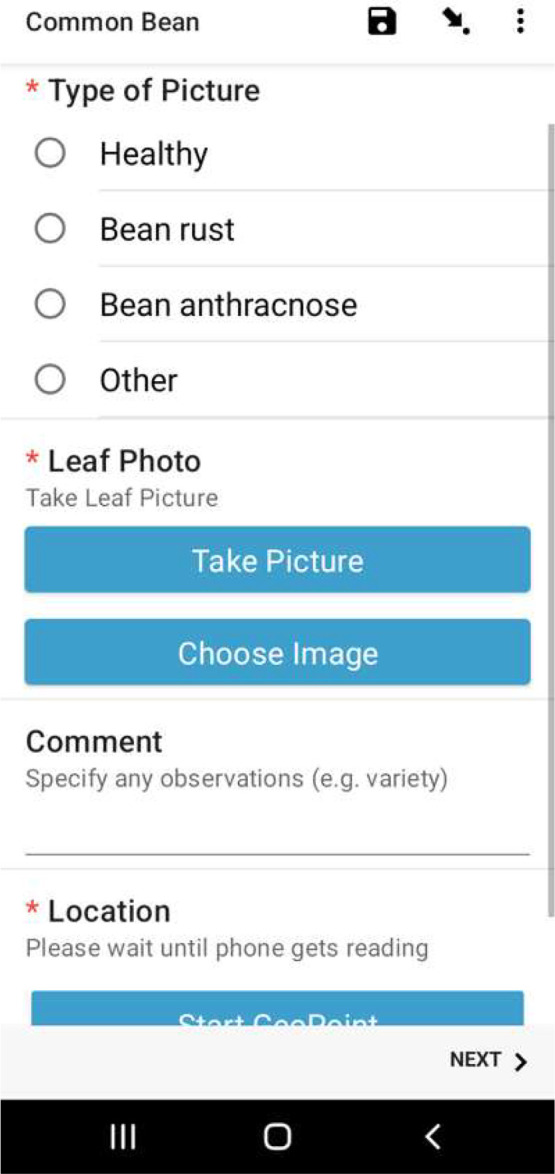
ODK form for collecting image data.

The dataset comprises common bean leaf images collected through a joint effort involving researchers and collaborators working in Northern Tanzania. Image acquisition was conducted using Samsung Galaxy M13 smartphones equipped with 13-megapixel cameras, with the Open Data Kit (ODK) application deployed to enable structured data capture in field settings (Fig. 1).

Data were collected over a one-year period, from January to December 2024, across three regions i.e., Arusha, Kilimanjaro and Manyara. These regions were selected based on their importance in common bean production and the observed prevalence of key diseases affecting the crop. The data collection process involved research assistants and smallholder farmers to ensure representation of real farming conditions. Images were primarily captured in situ under natural field conditions to reflect realistic disease presentation. Image resolution was determined by the device settings and maintained consistently across the data collection process. In some cases, multiple images of the same plant or leaf may be present due to repeated sampling under different angles or conditions.

To ensure data quality and consistency, all collected images were reviewed and validated by agricultural extension officers and plant pathologists. The annotation process followed a structured workflow in which each image was independently assessed by at least two experts based on visible disease symptoms. A standardized symptom checklist, derived from established field diagnostic guidelines for common bean diseases was used to guide labeling decisions. In cases of disagreement, images were jointly reviewed and discussed until a consensus was reached. Where necessary, uncertain samples were excluded from the final dataset to maintain label reliability. This multi-step validation process helped ensure the accuracy and consistency of the assigned labels.

### 4.2 Data preprocessing

**Fig. 2:**
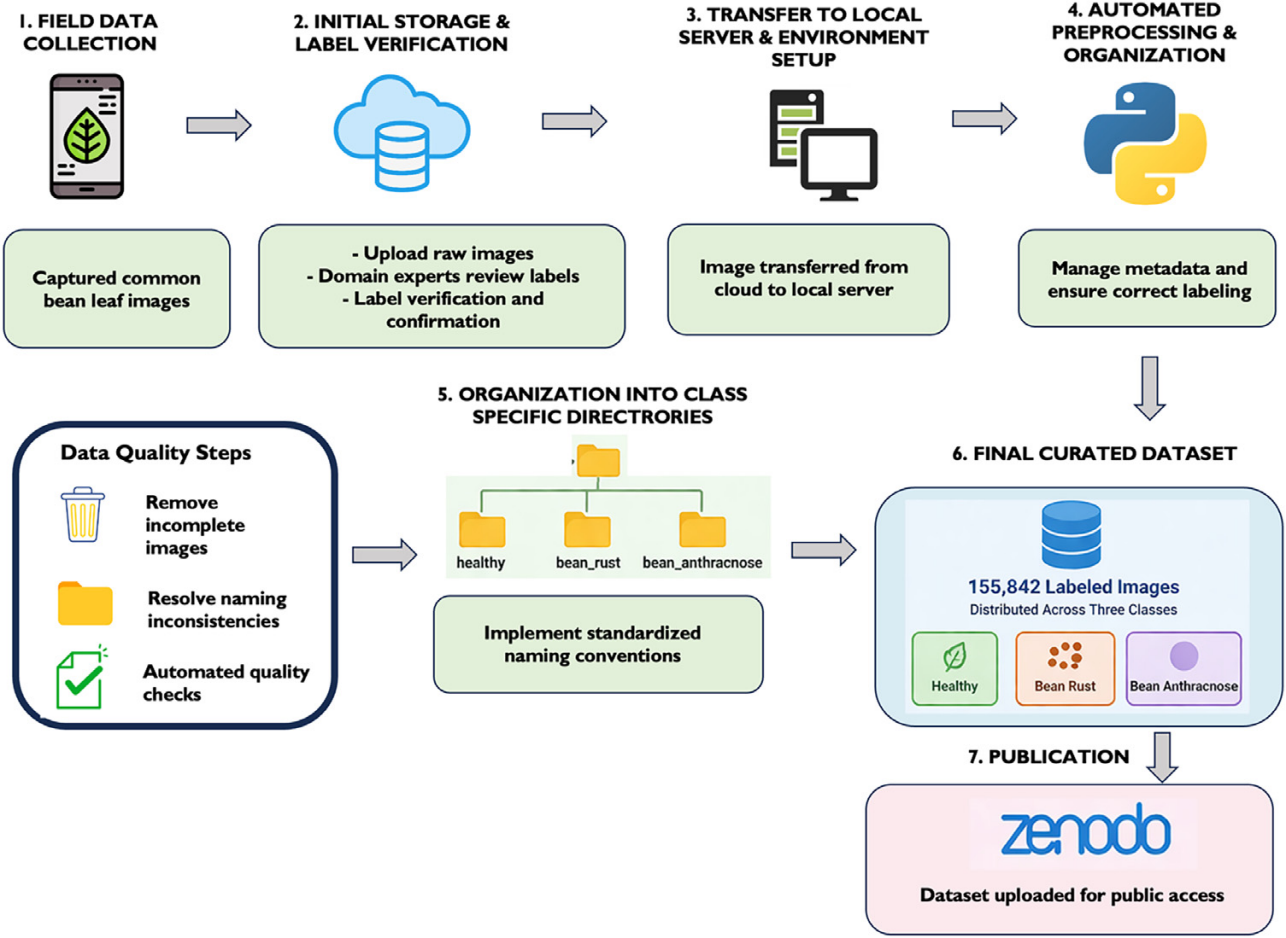
Data preprocessing pipeline of the common bean imagery leaves.

Following field collection, the common bean leaf images were subjected to a series of preprocessing steps prior to publication in an open-access repository (Fig. 2). The raw data captured were initially stored in cloud-based spreadsheets, where labels provided by field officers and domain experts were reviewed, verified and confirmed.

Preprocessing involved transferring the images from the cloud to a local server environment, where automated scripts developed in Python (within a Conda environment) were used to organize and prepare the dataset. Libraries such as Pandas were utilized to manage metadata and ensure correct mapping between images and their labels as part of the preprocessing and organization stage.

Several data quality challenges were addressed during preprocessing. These included handling incomplete or corrupted image files, inconsistencies in file naming and mismatches between image files and their corresponding metadata. Automated checks were applied to detect and remove unusable images, while standardized naming conventions were introduced to ensure consistency and traceability. A structured renaming process was implemented, linking each image to its metadata (e.g., GPS information) through generated reference files.

The processed images were then organized into class-specific directories namely healthy, bean rust and bean anthracnose, based on the validated annotations. The final curated dataset comprises 155,842 labeled images distributed across the three classes. The dataset was uploaded to a publicly accessible repository for wider use [20].

The preprocessing steps were designed to ensure data quality, consistency and usability for downstream applications such as crop disease detection. These steps focused on data cleaning, validation and structured organization rather than model development, in line with the objective of preparing a reliable dataset for reuse. Particular care was taken throughout preprocessing to maintain data integrity and ensure reliability for downstream computer vision applications.

## Limitations

The dataset consists of common bean leaf images collected from selected regions in Northern Tanzania specifically Arusha, Kilimanjaro and Manyara. While these areas are important for bean production, the geographical coverage is limited and may not fully represent conditions in other parts of the country. In addition, the dataset focuses on only two major diseases i.e., bean rust and bean anthracnose which are known to significantly affect productivity in the selected regions. Other diseases that may impact common bean production are not included.

Although expert validation and quality control procedures were applied during annotation and preprocessing, the possibility of residual labeling errors cannot be completely eliminated. Furthermore, the dataset may contain variability associated with environmental conditions during image acquisition, differences in disease progression and changes in leaf appearance such as partial drying after collection. Potential sampling biases related to specific farming practices or locations may also exist.

To ensure high data quality, only images captured under suitable lighting conditions were retained, while those taken in poor lighting environments were excluded. As a result, the dataset may have limited representation of challenging real-world conditions. Future work could extend the dataset by including additional disease categories and broader geographical coverage to improve dataset diversity and applicability.

## Ethics Statement

Data were gathered from farms following a formal consent procedure. Participating farmers signed consent forms granting permission for data collection to take place on their fields.

## CRediT Author Statement

**Neema Mduma:** Conceptualization, Methodology, Software, Validation, Formal analysis, Investigation, Resources, Data Curation, Writing - Original Draft, Writing - Review & Editing, Visualization, Supervision, Project administration, Funding acquisition; **Hudson Laizer:** Conceptualization, Methodology, Writing - Review & Editing, Supervision, Project administration.

## Data Availability

ZenodoA Labeled Dataset of Healthy and Diseased Common Beans from Tanzania (Original data)
